# Impact of meteorological parameters in fruit splitting of Daisy mandarin under subtropical conditions

**DOI:** 10.3389/fpls.2025.1700833

**Published:** 2025-12-15

**Authors:** Komalpreet Kaur, Monika Gupta, H. S. Rattanpal, Shubham Anand, Gurteg Singh, Dimpy Raina

**Affiliations:** 1 Department of Fruit Science, Punjab Agricultural University, Ludhiana, India; 2 Department of Climate Change and Agricultural Meteorology, Punjab Agricultural University, Ludhiana, India

**Keywords:** cracking, mandarin, meteorological factors, rainfall, temperature

## Abstract

Fruit splitting is a critical physiological disorder adversely affecting the fruit quality and marketability of citrus fruits, with its incidence closely associated with prevailing meteorological conditions. This study investigates the influence of weather parameters on fruit splitting in *Daisy Mandarin*. For this study, daily meteorological data on temperature, relative humidity and rainfall were obtained from the Agrometeorological Observatory, Punjab Agricultural University (PAU), Ludhiana. The results of this study revealed that a significantly higher incidence of fruit splitting was recorded in 2022 as compared to 2021, with key weather parameters playing a major role. Correlation and regression analyses identified that the maximum and minimum temperatures, morning and evening relative humidity and rainfall as the most influential factors. Higher temperatures accelerated the fruit growth, causing structural stress and subsequent splitting, while high humidity increased peel moisture content, making fruits more susceptible. Additionally, heavy rainfall following dry periods further intensified splitting due to rapid water uptake and internal pressure changes. These findings highlight the complex interactions between the meteorological parameters and underscore the need for adaptive management strategies to reduce fruit splitting under variable weather conditions.

## Introduction

Citrus is among the most significant fruit crops globally, cultivated widely in tropical and subtropical climates (Kaur, 2023; Bagri et al., 2021). In terms of area and production, citrus ranks third worldwide, following mango and banana. In India, citrus is widely cultivated on a 14.4 million hectares, contributing to an annual production of 150 million tons (FAOSTAT, 2023).

In India, mandarins are highly popular in India due to their easy-to-peel nature, high juice content and excellent taste. The northwestern part of India is globally renowned for Kinnow mandarin production, contributing significantly to citrus production (Kaur et al., 2024a, 2025a; Kaur 2023). Attempts have been made to introduce new citrus cultivars from the USA to the northwest region of India for increasing yield, good quality and early fruit maturity to enhance the availability period of citrus fruits for juice and fresh consumption (Kaur et al., 2024b, 2024c). Among them, Daisy mandarin (*Citrus reticulata* Blanco) was found promising, considering its early maturity and high fruit quality and was introduced in India to diversify the Kinnow mandarin (Kaur et al., 2024b, 2024d). Another major aspect of introducing this fruit was to increase the market period of mandarin in Punjab from November to February, as it is an early-bearing cultivar (Kaur et al., 2024a, 2024b; Dhillon et al., 2016). However, apart from its commercial potential, Daisy mandarin faces several challenges, the most notable being fruit splitting, a physiological disorder that severely effecting the fruit quality and marketability (Kaur et al., 2024c; Xylia et al., 2021).

Fruit splitting, a pre-harvest physiological disorder characterized by meridional fissures originating at the stylar end and extending towards the equatorial zone of the fruit. This results in the visible peel cracks, increased water loss and heightened susceptibility to pathogen invasion (Yu et al., 2020; Kaur et al., 2024e). The disorder affects more than 60% of global citrus production (Chabbal et al., 2020), significantly reducing the fruit quality and marketability, leading to economic losses for growers (Kaur et al., 2024e; Abdollah, 2015; Kaur et al., 2025b). The incidence of fruit splitting varies across different citrus varieties and regions, but is primarily influenced by several abiotic factors such as weather conditions, rootstock selection, tree age, and orchard management practices (Spade et al., 2024; El-Sayed, 2016). Additionally, nutrient imbalances, particularly deficiencies in calcium (Ca), boron (B), magnesium (Mg), and potassium (K), contribute to increased fruit-splitting rates. An excessive supply of nitrogen (N), which exerts pressure on the fruit’s epidermis, further exacerbates this problem (Kaur et al., 2023).

Climate variability plays a pivotal role in fruit splitting, with weather conditions such as high temperatures, fluctuating humidity and irregular rainfall are identified as key contributors (Mesejo et al., 2024). High temperatures during critical growth stages accelerate the fruit expansion, increasing internal stress and leading to splitting (Li, 2009). Low humidity and high evapotranspiration exacerbate water loss, making fruit peels more prone to splitting. Sudden changes in temperature, particularly during the fruit coloring stage, disrupt the elasticity of peel, contributing to fruit splitting (Adiba et al., 2024). Rainfall dynamics are especially critical. Heavy rainfall during the maturation phase or following prolonged drought induces rapid fruit expansion, causing structural stress and splitting. This abrupt change in moisture availability affects the fruit turgor pressure, intensifying the splitting risks. Intensity of light further influences the water uptake and peel stress, with higher light intensity linked to increased fruit splitting incidence (Butani et al., 2019).

Although fruit splitting has been widely studied in various citrus species, but there is a distinct lack of research specifically addressing the meteorological factors contributing fruit splitting in Daisy mandarin. This cultivar, is known for its early maturity and high fruit quality, may respond differently to climatic fluctuations compared to other mandarins due to its unique physiological and phonological traits. Understanding how the specific weather variables such as temperature extremes, rainfall patterns and humidity changes affect fruit splitting in Daisy mandarin remains a significant knowledge gap. Therefore, this study aims to evaluate the impact of key weather parameters on fruit splitting in Daisy mandarin, providing essential insights for developing preventive strategies and improving the orchard management practices to enhance the fruit quality and marketability.

## Materials and methods

### Experimental site

The study was conducted during 2021 and 2022 growing seasons at College Orchard, Department of Fruit Science, Punjab Agricultural University (PAU), Ludhiana. Being part of India’s trans-Gangetic agroclimatic zone, Ludhiana is situated at an altitude of 247 meters above mean sea level in the central plain region of Punjab at 30°54’ N latitude and 75°48’ E longitude. In general, climatic conditions here are subtropical and semi-arid. December and January are coldest months of region, while May and June are hottest months. Rainfall during the monsoon season occurs between the month of June to September. The average maximum and minimum temperatures for the year are 29.8°C and 16.7°C, respectively. Sometimes, during winter season, frost occurs in December and January when the minimum temperature drops even below 0.0°C and during the summer season, the temperature may exceed 45°C. The average annual rainfall is 760 mm, out of this 75 to 80 per cent is received between June to September i.e. monsoon season. The mean maximum and lowest temperatures exhibit significant variances from the normal over the summer and winter seasons.

### Planting material

A total of ten plants were included for both the healthy and the split-fruited tree groups. Measurements were collected from each plant, and the data were subsequently analyzed based on the average values obtained across these trees. Accordingly, the study utilized ten trees per group, with sampling conducted at a consistent frequency throughout the observation period. The experimental plants were planted at a spacing 6 m × 3 m apart and were managed according to standard cultural practices as prescribed by Package & Practices of fruit crops. Punjab Agricultural University, Ludhiana (Anonymous, 2021). These eight-year-old experimental plants were selected based on their uniformity in size, shape, age and vigor. Consistent cultural practices, including irrigation, fertilization, manuring and plant protection measures were employed throughout the study

### Meteorological data collection

**Figure 1:**
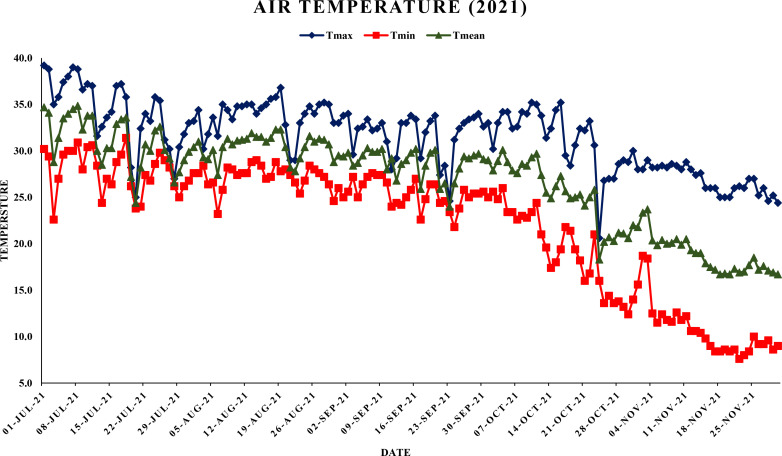
Maximum, minimum and mean air temperature during year 2021.

**Figure 2:**
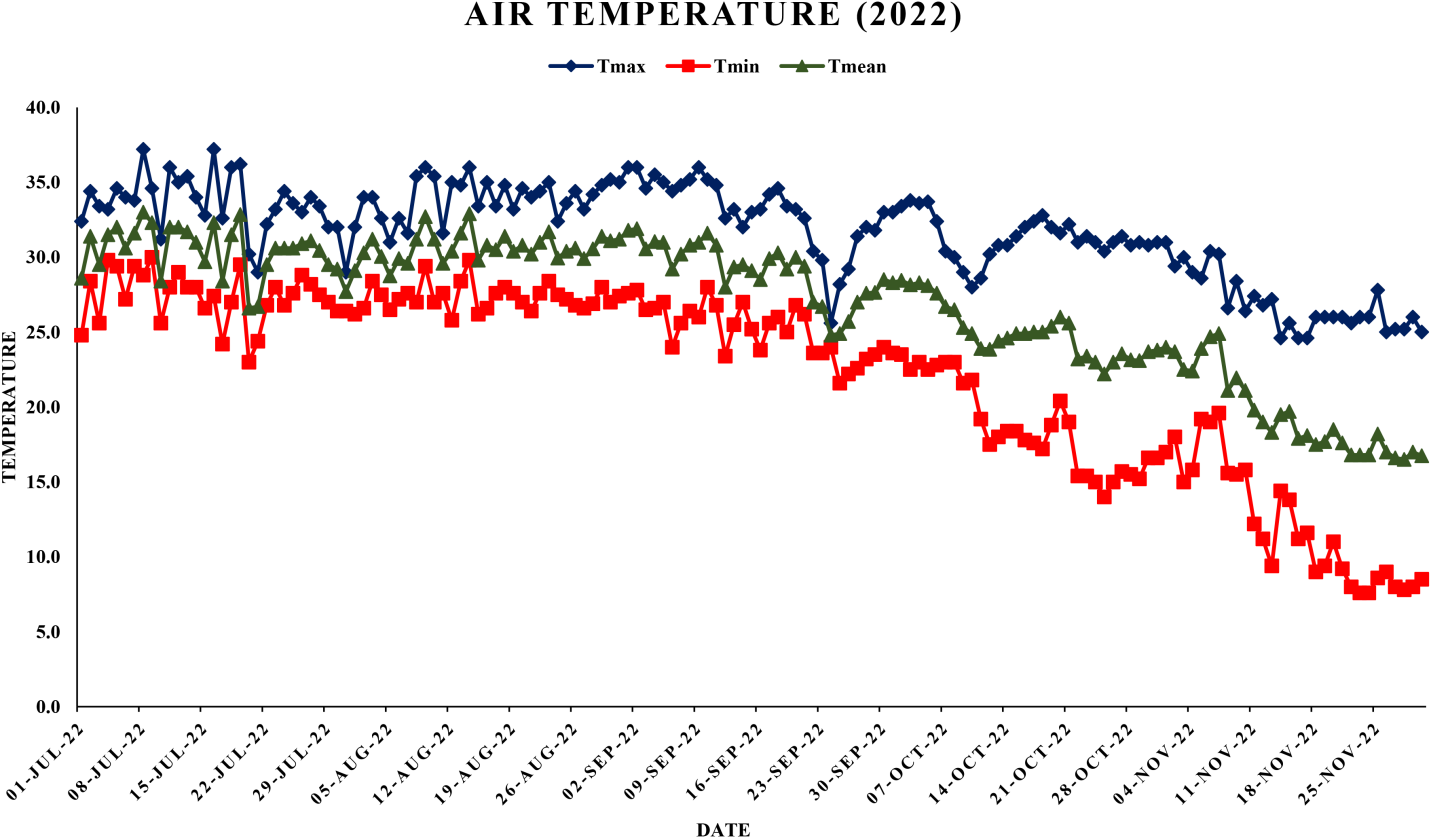
Maximum, minimum and mean air temperature during year 2022.


The meteorological data viz., maximum and minimum temperatures, morning and evening relative humidity and rainfall was collected from Agrometeorological observatory located at Punjab Agricultural University, Ludhiana as shown in **Figures 1**, **2**. Weather data were averaged on a weekly basis to minimize short-term fluctuations and to provide a representative summary of environmental conditions during the study period. Daily diurnal range of temperature was computed using formula:

Diurnal range of temperature= Maximum temperature−Minimum temperature

Thermal humid index (THI) was calculated using the following formula:


$$\text{Thermal humid index }(\text{THI})=\frac{\text{Mean temperature}}{\text{Mean relative humidity}}$$

### Observations recorded

**Figure 3:**
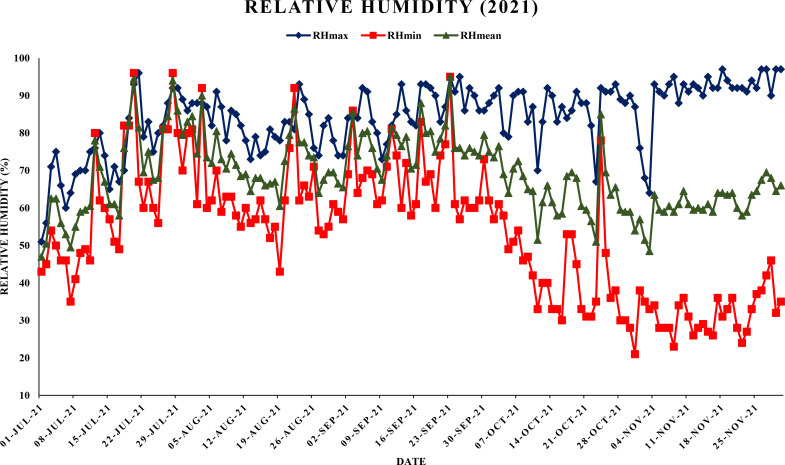
Maximum, minimum and mean relative humidity (%) during year 2021.

**Figure 4:**
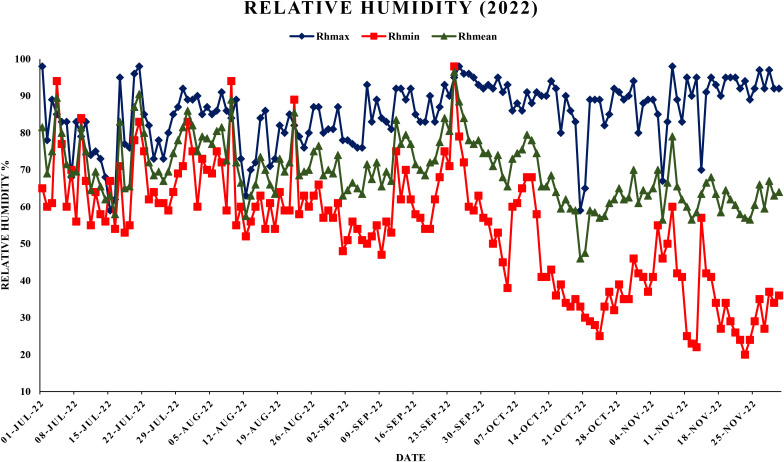
Maximum, minimum and mean relative humidity (%) during year 2022.

**Figure 5:**
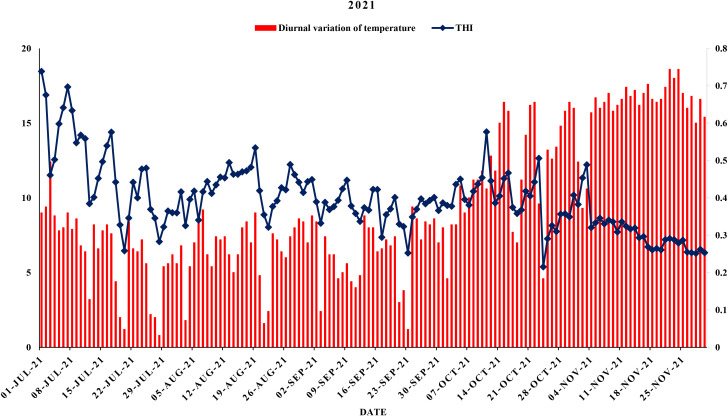
Diurnal variation of temperature and thermal humid index during year 2021.

**Figure 6:**
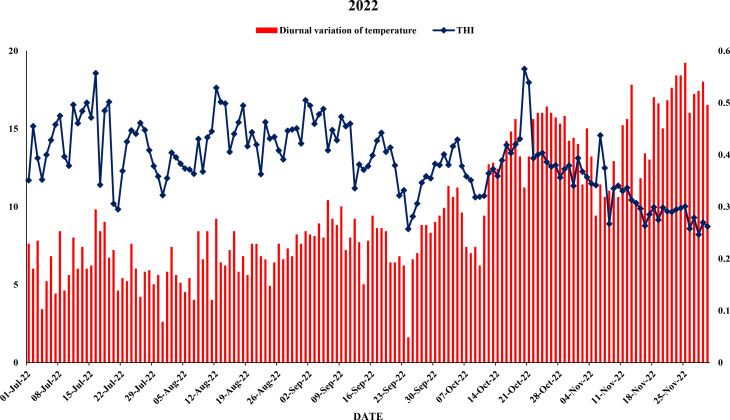
Diurnal variation of temperature and thermal humid index during year 2022.

The experiment was conducted in a randomized complete block design (RCBD) with three replications. The Daisy mandarin plants were selected for the study. The experimental trees were planted at a spacing 6 m × 3 m apart and were managed according to standard cultural practices as prescribed by Package of Practices for cultivation of fruit. Punjab Agricultural University, Ludhiana (Anonymous, 2021). These eight-year-old experimental plants were selected based on their uniformity in size, shape, age and vigor. To ensure uniformity and minimize experimental error, five branches representing different orientations (north, south, east, west and center) were selected from each tree in every replication. These branches were chosen to ensure uniform representation of environmental exposure and growth conditions. **Figures 3**–**6**,

### Measurement of Fruit splitting percentage (%)

The number of fruits on each tree was recorded periodically from the 90 days after fruit set (DAFS) until the harvest (210 DAFS), during both the years 2021 and 2022. The assessment was carried out at regular monthly intervals. To evaluate the extent of fruit splitting, the number of split fruits was counted at each interval and the fruit splitting percentage was calculated by dividing the number of split fruits by the total number of fruits on the tree. The following formula was used to calculate the fruit splitting percentage:


$$Fruit\;splittitng\;(\%)=\frac{Number\;of\;splitted\;fruits\;}{Total\;number\;of\;fruits\;}\times 100$$

### Measurement of peel thickness (mm)

The thickness of Daisy mandarin fruits was assessed by using a digital Vernier caliper’s (Mitutoyo, Japan) on both sides of the fruits, and mean data were expressed in millimeter (mm). For the measurement of peel thickness, the peel of the fruits was separated by hand peeling.

### Measurement of peel weight (g)

The weight of the fruit peel was measured by the using an electronic weighing balance (EK6100I A&D, Japan). The average weight of the fruit peel was calculated and expressed in grams.

### Measurement of water content in peel, pulp and fruit (%)

For the measurement of water content of the peel, weight and fruit, the fresh weight of fruit peel, pulp and fruit was recorded and subsequently, they were placed in an oven at 65°C for drying. After 48 hours, the dry weights were recorded.

### Statistical analysis

The experimental data were examined by using the SAS software (version 93, SAS Institute Inc. USA). Subsequently, correlation and regression analysis were performed to determine the relationship between meteorological parameters and fruit splitting severity. Additionally, weather-based regression models were developed using R software to predict fruit splitting severity based on meteorological conditions.

## Results

### Peel thickness (mm)

The results revealed a consistent trend in peel thickness in Daisy mandarin fruits over the different developmental stages from 90 DAFS to 210 DAFS (**Table 1**). In both years, 2021 and 2022, the maximum peel thickness was recorded at the initial sampling date i.e. 90 DAFS, followed by a gradual decline until the onset of fruit maturity till 210 DAFS. Data showed that the maximum peel thickness (4.59 mm and 4.57 mm) was recorded in 2021 and 2022 at first sampling date (90 DAFS) and thereafter started declining till the onset of fruit maturity. A progressive decline in peel thickness was recorded as the fruit approached maturity, with the minimum peel thickness was recorded at 210 DAFS (1.62 mm and 1.44 mm).

**Table 1:** Fruit characteristics of Daisy mandarin during the 2021 and 2022 growing seasons.

Fruit characteristics	Peel thickness (mm)		Peel weight (g)		Pulp weight (g)		Water content of pulp (%)		Water content of fruit (%)		Splitting (%)	
Year	2021	2022	2021	2022	2021	2022	2021	2022	2021	2022	2021	2022
90 (DAFS)	4.59	4.57	12.42	11.76	25.99	29.23	64.77	65.9	70.81	71.22	3.48	4.37
120 (DAFS)	4.2	4.13	15.33	13.36	33.57	53.14	67.34	68.83	72.96	73.13	3.46	3.71
150 (DAFS)	3.24	3.17	28.41	28.06	33.05	54.6	79.41	79.49	76.41	76.64	2.7	2.91
180 (DAFS)	1.98	1.67	31.82	29.29	54.75	79.14	79.97	80.05	76.01	77.57	1.67	2.09
210 (DAFS)	1.62	1.44	36.85	36.69	74.81	137.04	86.49	87.46	80.15	79.39	1.37	1.45

### Peel weight (g)

The data presented revealed a significant variations in peel weight of Daisy mandarin fruits during the developmental stages from 90 DAFS to 210 DAFS during 2021 and 2022 (**Table 1**). A significant reduction in peel weight (11.76 g) was noticed at 90 DAFS in 2022 as compared to 2021 (12.42 g), potentially reflecting the influence of weather conditions. Subsequently, the peel weight showed an increasing trend from 90 DAFS to 210 DAFS in 2021, while this trend was less pronounced in 2022.

### Pulp weight (g)

The results showed a significant relationship between days after fruit set and the pulp weight of Daisy mandarin fruits (**Table 1**). It was observed that the pulp weight was enhanced significantly from 90 DAFS to 210 DAFS during both 2021 and 2022, demonstrating the natural progression of fruit development. At 90 DAFS the pulp weight in 2022 was found significantly higher (29.23 g) than in 2021 (25.99 g), indicating an interannual variability influenced the environmental conditions.

### Water content of pulp

The water content of the pulp in Daisy mandarin fruits exhibited the significant variability during developmental stages i.e. from 90 to 210 DAFS during 2021 and 2022 (**Table 1**). It was noticed that at 90 DAFS, the water content was found significantly higher (65.90%) during 2022 as compared to 2021 where lower amount of water content was recorded (64.77%).

### Water content of fruit

The results related to water content in Daisy mandarin fruits exhibited significant variability across different development stages from 90 to 210 DAFS during 2021 and 2022 (**Table 1**). A significant year-to-year to variation was observed, with higher water content was recorded in 2022. At 90 DAFS, the water content was higher in 2022 (71.22%) as compared to 2021 (70.81%), reflecting an influence of environmental factors. The highest water content of the fruit was observed at the early stages of development from 90 DAFS to 150 DAFS, followed by gradual decline as fruit matured. By the 210 DAFS, water content decreased from 80.15% in 2021 to 79.39% in 2022.

### Fruit splitting severity

Fruit splitting is a significant physiological disorder that can adversely affect the quality and marketability of citrus fruits, including Daisy mandarin (**Table 1**). The results revealed notable variations in fruit splitting incidence between the two years and across the developmental stages. The fruit splitting percentage for Daisy mandarin fruits during the years 2021 and 2022 were recorded and throughout the period of development from 90 DAFS to 210 DAFS, respectively. From the given data, it was noted that the higher incidence of fruit splitting was recorded in 2022 as compared to 2021, suggesting an influence of interannual variability in weather conditions. At 90 DAFS (July), the peak fruit splitting percentage was recorded and then declined. The maximum fruit splitting (4.16% and 3.48%) was recorded followed by 120 DAFS (3.71% and 3.46%), in 2022 and 2021 at 90 DAFS, respectively, while fruit lowest fruit splitting was recorded at the 210 DAFS (1.45% and 1.37%) during 2022 and 2021, respectively.

### Meteorological parameters during the 2021 and 2022 growing seasons

**Figure 7:**
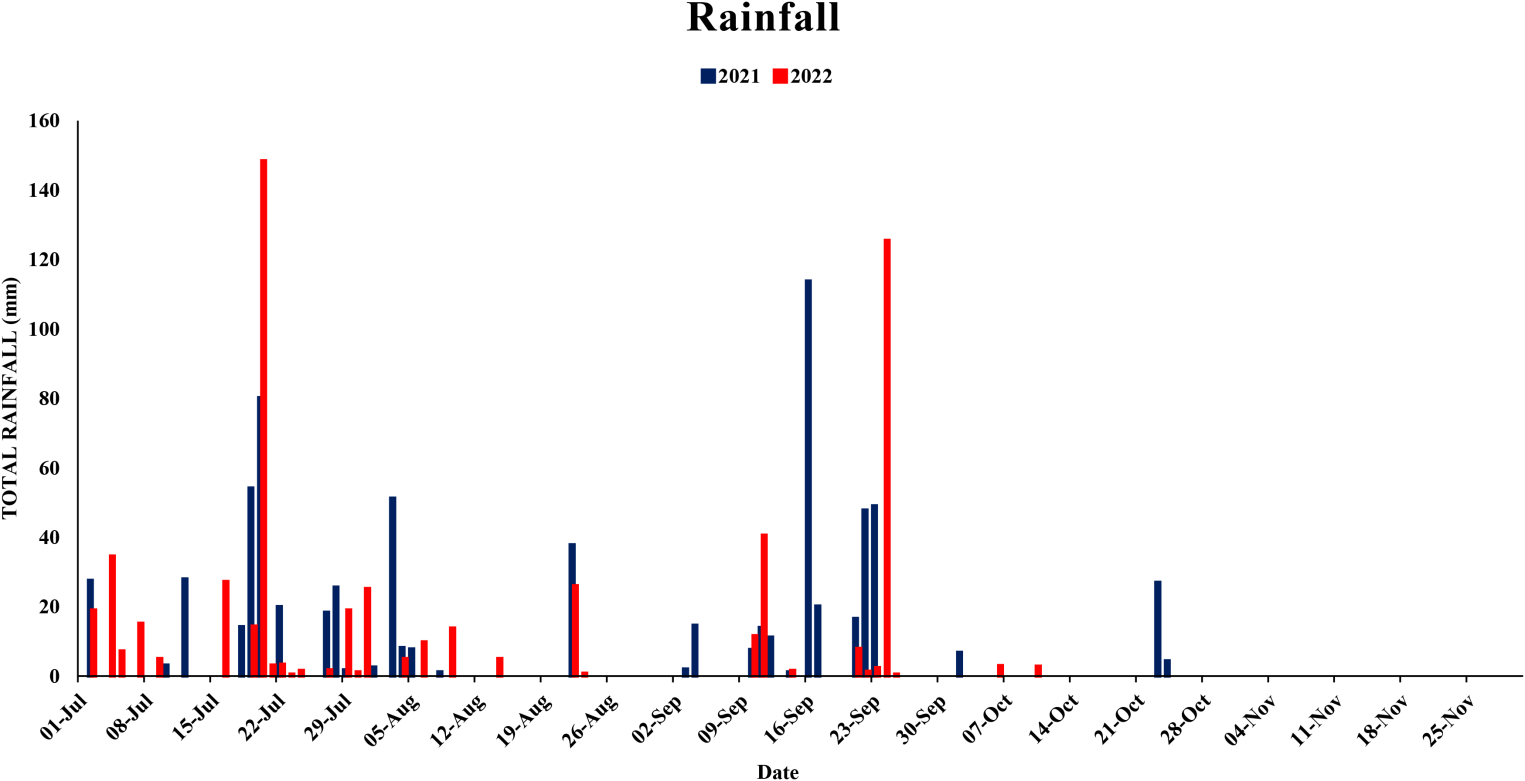
Total rainfall (mm) during years 2021and 2022.

This study analyzed the meteorological parameters influencing fruit splitting in Daisy mandarin during the 2021 and 2022 growing seasons (**Table 2**). Key variables such as temperature, relative humidity, rainfall, and the temperature-humidity index (THI) were examined across five critical stages: 90, 120, 150, 180, and 210 days after fruit set (DAFS). The findings highlight how environmental conditions directly affect physiological processes, leading to fruit splitting. At 90 DAFS, maximum temperature ranged from 25.0–39.2°C, while minimum temperature varied between 22.6–31.4°C during year 2021 (**Figures 1**, **2**). Relative humidity was high, with morning values between 51–96% and evening values from 35–96% (**Figures 3**, **4**). Rainfall totaled 271.2 mm, contributing to a fruit splitting percentage of 3.48%. A diurnal temperature variation of 0.8–12.4°C and THI values of 0.3–0.7 indicated moderate thermal stress. By 120 DAFS, rainfall decreased to 107.6 mm, and maximum temperature stabilized at 29.0–36.8°C. Relative humidity values remained high (morning: 73–93%, evening: 43–92%), while the THI reduced to 0.3–0.5, lowering thermal stress. The splitting percentage remained nearly constant at 3.46%. At 150 DAFS, high rainfall (295.8 mm), with temperatures ranging from 24.6–34.0°C (maximum) and 21.8–27.6°C (minimum). A narrower diurnal temperature variation (1.2–9.4°C) and THI values of 0.25–0.4 corresponded to a reduced splitting percentage of 2.7%. By 180 DAFS, reduced rainfall (37.6 mm) and cooler conditions (maximum: 20.6–35.2°C, minimum: 12.4–26.0°C) led to a further decline in splitting percentage (1.67%). At 210 DAFS, the dry end-of-season conditions (no rainfall) and greater diurnal variations (9.3–18.6°C) were associated with the lowest splitting percentage (1.37%) (**Figures 5**, **6**). Higher temperature enhances the fruit expansion, which cause mechanical stress that influences the peel to splitting (Drogoudi et al., 2021; Xylia et al., 2021). Similarly, increased relative humidity during critical growth stages affects moisture content, making the fruit peel more susceptible to splitting (Yu et al., 2020). Heavy rainfall during the July period in 2022 likely exacerbated fruit splitting by causing sudden and excessive water uptake, leading to an imbalance between fruit turgor pressure and peel elasticity (Butani et al., 2019). The interaction between high temperature and humidity is particularly detrimental during early fruit development. Rapid fruit expansion under such conditions may surpass the tensile strength of the peel, resulting in meridional fissures (Abd & Rahman, 2010). The significant rainfall events recorded in July of 2022 likely intensified this phenomenon by introducing abrupt changes in soil moisture, further exacerbating the splitting incidence. The observed trends in Daisy mandarin splitting are consistent with reports on other citrus species. For instance, Li et al. (2009) emphasized the role of fluctuating temperature and moisture levels in inducing fruit cracking in mandarins. Similarly, Yu et al. (2020) highlighted the importance of maintaining balanced irrigation practices to mitigate splitting during sensitive growth phases. The 2022 season exhibited distinct climatic variations. At 90 DAFS, higher rainfall (323.8 mm) combined with elevated temperatures (maximum: 29.0–37.2°C, minimum: 23.0–30.0°C) and relative humidity (morning: 59–98%, evening: 53–94%) led to the highest splitting percentage of 4.37%. At 120 DAFS, reduced rainfall (59.2 mm) and moderated temperatures (maximum: 31.0–36.0°C, minimum: 25.8–29.8°C) resulted in a splitting percentage of 3.71%. By 150 DAFS, rainfall was 190.1 mm, with temperatures stabilizing between 25.6–36.0°C (maximum) and 21.6–28.0°C (minimum) (**Figure 7**). These conditions reduced the splitting percentage to 2.91%. By 180 DAFS, low rainfall (5.4 mm) and lower humidity levels were observed, coinciding with a splitting percentage of 2.09%. At 210 DAFS, dry conditions persisted (no rainfall), with greater diurnal variations (9.4–19.2°C) contributing to the lowest splitting percentage (1.45%).

**Table 2:** Meteorological parameters influencing fruit splitting in Daisy mandarin during the 2021 and 2022 growing seasons.

Year	Days after fruit setting (DAFS)	T_max_	T_min_	T_mean_	Rh_max_	Rh_min_	Rh_mean_	Rf	THI	Splitting percentage (%)
2021	90	34.3	27.8	31.1	75.8	61.9	68.9	271.2	0.5	3.48
	120	33.8	27.2	30.5	82.0	62.6	72.3	107.6	0.4	3.46
	150	31.8	25.4	28.7	86.4	68.1	77.3	295.8	0.4	2.7
	180	31.3	19.5	25.4	86.5	42.8	64.7	37.6	0.4	1.67
	210	26.8	10.7	18.8	90.6	32.1	61.3	0.0	0.3	1.37
2022	90	33.5	27.3	30.4	80.8	66.4	73.6	323.8	0.4	4.37
	120	34.0	27.3	30.7	81.6	63.8	72.7	59.2	0.4	3.71
	150	33.0	25.2	29.1	87.4	61.0	74.2	190.1	0.4	2.91
	180	31.4	18.8	25.1	87.5	42.8	65.1	5.4	0.4	2.09
	210	26.9	12.2	19.5	89.7	36.0	62.9	0.0	0.3	1.45

The analysis revealed that early-season rainfall and high humidity, particularly at 90 DAFS, significantly influenced fruit splitting. Excessive rainfall during this stage increased fruit expansion rates, making the rind susceptible to cracking under fluctuating temperatures. For instance, higher rainfall in 2022 (323.8 mm) at 90 DAFS resulted in a splitting percentage of 4.37%, compared to 3.48% in 2021. As the growing season progressed, a reduction in rainfall and thermal stress, reflected by declining THI values, contributed to a consistent decrease in fruit splitting percentages. Low rainfall and stable temperatures at 210 DAFS in both years created conditions favorable for fruit integrity, resulting in the lowest splitting percentages (1.37% in 2021 and 1.45% in 2022).

## Discussion

The observed decline in peel thickness over time can be attributed due to the physiological changes associated with the fruit maturation, particularly the redistribution of cell wall components and the increasing elasticity of peel tissues. These results are align with earlier studies highlighting the peel thickness as a key determinant of fruit splitting resistance in citrus cultivars (El-Sayed, 2016; Yu et al., 2020). Prevailing weather conditions during the study period played a crucial role in influencing fruit splitting. A significant correlation was observed between the peel thickness and fruit splitting. Fruits with thicker peels exhibited a lower incidence of splitting as compared to those with thinner ones (Kaur et al., 2024a). The protective role of thicker peels can be explained by their ability to resist internal turgor pressure excreted by the juice sacs, especially during the periods of rapid fruit growth or environmental stress i.e. high temperature, humidity fluctuation or heavy rainfall (Dovjik et al., 2024; Li, 2009). In contrast, the fruits having thinner peels lack the mechanical strength to withstand these pressures making them more susceptible to fruit splitting. As the thinner peel is less capable of accommodating the internal volume changes mainly caused by rapid uptake of water or uneven cell expansion, leading to the development of cracks or meridional fissures (Butani et al., 2019).

At 90 DAFS, a notable reduction in peel weight was recorded in 2022 as compared to 2021, and this decrease was strongly associated with a higher incidence of fruit splitting. The decrease in peel weight was observed at the early stage of fruit development (90 DAFS) in 2022 can be attributed due to the adverse environmental conditions, including the rainfall, high evapotranspiration and fluctuations in temperature. Such conditions are known th hinder the uniform vegetative and reproductive growth, thereby influencing the peel formation and structural integrity. These observations are align with the previous findings that emphasize that the sensitivity of citrus peel development to environmental stressors, which ultimately affects the overall fruit quality (Chabbal et al., 2020; Yu et al., 2020). There was a strong positive correlation was noticed between the peel weight and the incidence of fruit splitting. Fruits having a higher peel weight exhibited the lower fruit splitting severity as recorded in 2021, while those fruits having reduced peel weights in 2022 demonstrated a higher susceptibility to splitting. Thinner peels provide a reduced mechanical strength and are less capable of accommodating the internal osmotic pressure during the periods of rapid growth or environmental instability (El-Sayed, 2016). The elevated fruit splitting observed in 2022 can therefore be attributed to erratic weather conditions such as excessive rainfall or sudden fluctuations in temperature, which can increase the internal turgor pressure beyond the peel’s structural capacity, resulting in rupture. These results support the earlier studies that highlight the critical role of environmental conditions in determining the peel development and fruit splitting dynamics (Li, 2009; Butani et al., 2019).

The notable increase in pulp weight recorded in 2022, can be attributed due to the favorable growing conditions including the consistent rainfall, optimal temperatures and adequate humidity during critical developmental stages. In contrast, the decreased pulp weight observed in 2021 likely resulted from suboptimal environmental weather conditions, including inadequate rainfall and extreme temperature fluctuations, which may have restricted cellular expansion and solute accumulation within the fruit. These findings consistent with the previous findings that emphasize the strong influence of environmental factors on citrus fruit growth and pulp development (Butani et al., 2019; El-Otmani et al., 2011). A positive relationship was noticed between the increased the pulp weight and higher fruit splitting incidence. As pulp weight increases due to enhanced cellular expansion and solute accumulation, the resulting rise in internal turgor pressure imposes the mechanical stress on the peel. When the peel of the fruit lacks sufficient strength to accommodate this rapid internal expansion, fissures may form, leading to fruit splitting. This mechanism was particularly evident in 2022, when periods of heavy rainfall followed by dry spells likely accelerated uptake of water and pulp expansion, producing the alternating cycles of expansion and contraction that intensified the peel stress (Li et al., 2012). Although the increased pulp weight reflects the active cellular growth and contributes to improved fruit quality, excessive and rapid water-driven expansion increases susceptibility to splitting, especially under the fluctuating temperature and moisture conditions.

An increase in fruit water content was recorded in 2022, emphasizing the higher incidence of fruit splitting, thereby underscoring the critical influence of moisture dynamics on the fruit development and structural integrity. Increased water content enhances the internal turgor pressure, contributing to the greater fruit size and weight, however the excessive hydration simultaneously increase the risk of pee failure. These finding are consistent with previous reports highlighting the balance between fruit hydration and peel resilience in citrus species (El-Otmani et al., 2011; Yu et al., 2020). The observed differences in water content between 2021 and 2022, can be attributed due to the distinct weather conditions. During 2022, the occurrence of higher rainfall likely promoted the excessive water uptake by the developing fruit, leading to increased pulp moisture content. Although adequate moisture is essential for fruit growth, rapid or excessive water absorption can induced the physiological stress by elevating the internal pressure beyond the peel’s mechanical stress on the peel. Previous studies have shown that such instability disrupts the balance between the expanding internal tissues and the accommodating peel structure, often leading to fissures and fruit splitting.

The correlation between water content and fruit splitting in Daisy mandarin is multifaceted. Although higher water content generally enhances the fruit size, texture and sensory quality (El-Otmani et al., 2011. Yu et al., 2020). However, the excessive moisture within the fruit substantially increases the internal turgor pressure, thereby elevating the risk of fruit splitting. The markedly higher rainfall and relative humidity recorded in 2022 as compared to 2021 likely contributed to the elevated water content observed. Excessive water uptake during the key developmental stages amplifies the internal pressure, particularly when accompanied by the rapid fruit growth, which places the considerable stress on the peel and predisposes fruits with the thinner skins to fruit splitting. Apart from this, fluctuations in water availability intensify this vulnerability. Episodic heavy rainfall followed by dry spells can cause the rapid cycles of fruit swelling and contraction, creating the mechanical stress on the peel surface. Previous studies have reported the similar results that inconsistent moisture supply disrupts that balance between pulp expansions and peel elasticity, ultimately resulting in the structure failure (Li et al., 2012). This, while increased water content contributes positively to fruit quality parameters, excessive or unstable moisture conditions can compromise the peel integrity and significantly increase the incidence of fruit splitting.

The early development stage (90 DAFS) was the most critical period for fruit splitting as observed in both years. The high splitting rate occurred during this stage aligns with periods of rapid growth, which creates mechanical stress on the peel (Li et al., 2009). At this time, the fruit experiences an accelerated cell expansion and increase in internal turgor pressure, while the peel is still relatively thin and has a limited elasticity. As the internal osmotic pressure increases with rapid sugar and water accumulation, the turgor-driven the expansion of pulp tissues can exceed the mechanical strength of the peel, which leads to splitting. Conversely, the decline in splitting incidence at later stage i.e. 210 DAFS, may be attributed to a gradual thickening of the peel and reduced growth dynamics (El-Sayed, 2016). Due to an excessive increase in temperature, hot dry winds followed by a dry season and significant fluctuations in day-night temperatures, specifically when temperatures exceed 38°C combined with a relative humidity, have been found to favor the fruit cracking (Ikram et al., 2020). Sudden changes between day and night temperatures, cause the species more prone to fruit cracking (Fischer et al., 2021). Apart from this, fruit that expose to direct sunlight has been reported to increase the fruit surface temperature and evapotranspiration, resulting in a high moisture loss and a greater susceptibility to cracking. When the rehydration occurs, the inelastic peel cannot accommodate rapid tissue swelling, further increasing the susceptibility to fruit splitting.

### Correlation coefficients between fruit splitting (%) and meteorological parameters

**Figure 8:**
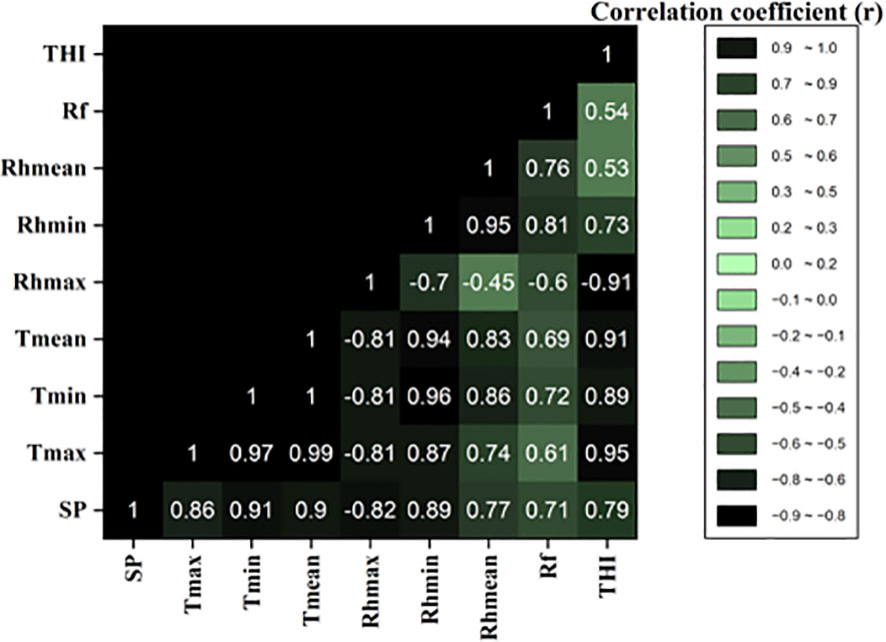
Correlation coefficients between fruit splitting (%) and meteorological parameters.

The correlation matrix reveals the relationships between meteorological parameters and fruit splitting (SP) in Daisy mandarin (**Figure 8**). Strong positive correlations were observed between fruit splitting (SP) and mean temperature (r = 0.90), minimum temperature (r = 0.91), and maximum temperature (r = 0.86). This indicates that higher temperatures, particularly during critical growth stages, significantly contribute to fruit splitting. Maximum relative humidity (Rh_max_) showed a moderate negative correlation with fruit splitting (r = -0.82), suggesting that lower Rh_max_ values are more favorable in reducing splitting. Minimum relative humidity (Rh_min_) displayed a strong positive correlation (r = 0.89) with SP, emphasizing the role of higher night time humidity in fruit stress. Mean relative humidity (Rh_mean_) had a moderately strong positive correlation (r = 0.77), reinforcing the combined impact of humidity on fruit splitting. Rainfall exhibited a moderate positive correlation with fruit splitting (r = 0.71), indicating that excessive rainfall during early fruit development stages exacerbates splitting by causing abrupt changes in fruit moisture content. The temperature-humidity index (THI) demonstrated a moderately strong positive correlation with SP (r = 0.79), signifying its effectiveness as an integrated metric for assessing thermal stress and predicting fruit splitting risk.

### Regression analysis

#### Temperature-humidity index vs peel thickness

**Figure 9:**
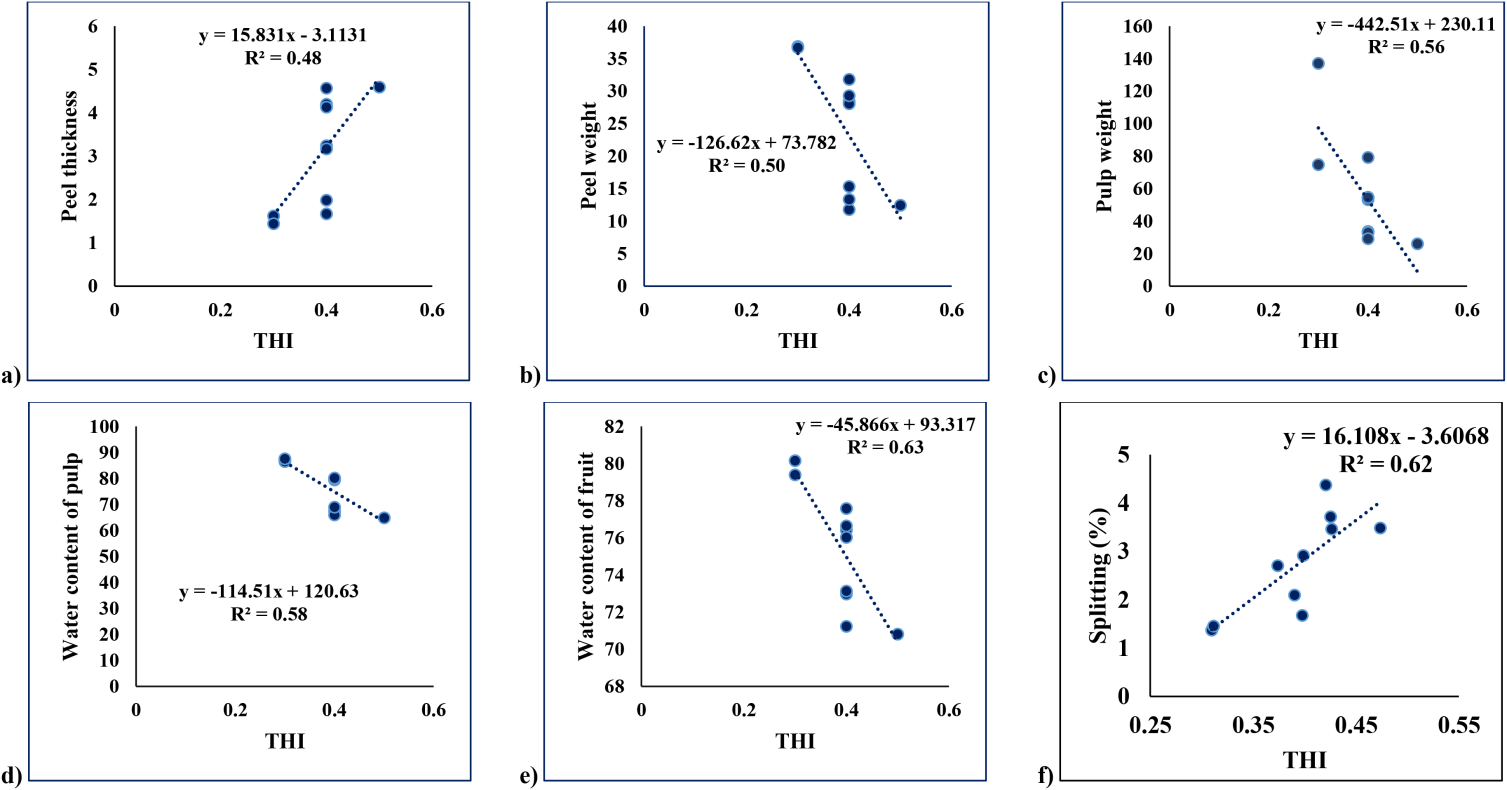
Regression analysis between THI and various fruit characteristics. **(a)**; Peel thickness, **(b)** Peel weight.; **(c)** Pulp weight; **(d)** Water content of pulp; **(e)** Water content of fruit; **(f)** Splitting (%).

The scatterplot illustrates a positive linear relationship between the Temperature-Humidity Index (THI) and peel thickness, as represented by the regression equation y = 15.831x - 3.1131 and R^2^ value of 0.48 (**Figure 9a**). The slope of the regression line suggests that peel thickness increases by approximately 15.83 units for every unit increase in THI. The R^2^ value indicates that 48% of the variation in peel thickness is explained by changes in THI, suggesting a moderate correlation. The observed trend highlights the potential influence of thermal and humidity stress on peel development, warranting further investigation into its physiological implications.

#### Temperature-Humidity Index vs peel weight

The scatterplot shows a negative linear relationship between the Temperature-Humidity Index (THI) and peel weight, as represented by the regression equation y = -126.62x + 73.782 and an R^2^ value of 0.50 (**Figure 9b**). The slope of the regression line indicates that peel weight decreases by approximately 126.62 units for every unit increase in THI. The R^2^ value suggests that 50% of the variability in peel weight is explained by THI. This inverse relationship highlights a potential adverse effect of higher THI on peel weight, likely due to environmental stress factors influencing fruit development.

#### Temperature-humidity index vs pulp weight

The scatter plot illustrates the relationship between pulp weight (y-axis) and THI (x-axis), where a negative correlation is observed. The regression line, defined by the equation y = -442.51x + 230.11, indicates that as THI increases, pulp weight decreases (**Figure 9c**). The coefficient of determination R^2^ = 0.56 suggests that approximately 56% of the variability in pulp weight can be explained by changes in THI. This moderately strong negative relationship highlights that higher THI values are associated with lower pulp weights.

#### Temperature humidity index vs water content of pulp

The plot explores the relationship between the water content of pulp and THI. Here, the regression line y = -114.51x + 120.63 also shows a negative relationship, meaning that as THI increases, the water content of the pulp decreases (**Figure 9d**). The R^2^ = 0.58 indicates that 58% of the variability in the pulp’s water content is explained by THI, showing a moderately strong correlation.

#### Temperature humidity index vs water content of fruit

The plot examines the relationship between the water content of fruit and the Temperature Humidity Index (THI). The regression equation y = -45.866x + 93.317 indicates a negative correlation, where higher THI values are associated with a decrease in the water content of the fruit (**Figure 9e**). The coefficient of determination R^2^ = 0.63 suggests that 63% of the variation in the fruit’s water content can be explained by changes in THI. This reflects a strong influence of environmental conditions on the fruit’s water retention capacity.

#### Temperature-humidity index vs fruit splitting percentage (%)

The scatterplot depicts the relationship between Temperature-Humidity Index (THI) and fruit splitting percentage (%) (**Figure 9f**). The regression line indicates a positive linear relationship between THI and fruit splitting. As THI increases, fruit splitting percentage also increases, suggesting that higher thermal and humidity stress levels contribute significantly to the incidence of fruit splitting.

The regression equation (y = 16.108x - 3.6068) quantifies the relationship.

Here:

y: Predicted splitting percentage (%)

x: THI

The slope of (16.108) implies that for every unit increase in THI, fruit splitting percentage increases by approximately 16.1%. The intercept (-3.6068) indicates the theoretical splitting percentage when THI is zero, though not practically relevant. The R^2^ value of 0.62 suggests that 62% of the variation in fruit splitting percentage can be explained by variations in THI. This indicates a moderately strong relationship, highlighting the importance of THI as a predictive factor for fruit splitting. The data points align reasonably well with the regression line, further supporting the positive correlation between THI and splitting percentage.

## Conclusions

This study demonstrates that the climatic variability caused a significant influence on the fruit splitting in Daisy mandarin. Among the studied factors, rainfall, temperature and relative humidity were identified as the major environmental factors affecting the fruit splitting. Increased temperatures and high humidity during the early stages of fruit development increased the splitting incidence, while the gradual stabilization of weather conditions in later stages contributed to a marked reduction in fruit spitting. The temperature-humidity index (THI) proved to be a reliable indicator of thermal stress and fruit splitting potential, highlighting its practical value for orchard management.

Overall, these findings emphasize the need for integrating the climatic monitoring and adaptive orchard practices, such as efficient irrigation scheduling and temperature moderation to minimize the fruit splitting and sustain the fruit quality. So that the future research should focus on developing predictive models based on weather indices to support early warning systems and inform management strate2gies for Daisy mandarin production under changing climatic conditions.

## Data Availability

The raw data supporting the conclusions of this article will be made available by the authors, without undue reservation.
